# A Novel Gamified Exercise Program Incorporating Stampede Training for Enhancing Functional Fitness, Physical Activity Levels, and Quality of Life in Community-Dwelling Older Adults: Randomized Parallel Exploratory Trial

**DOI:** 10.2196/73474

**Published:** 2025-11-03

**Authors:** Siao-Fen Huang, Ching-Wen Fan, Diana Khasna Nisrina, Tsai-Wei Lin, Wen Ching Huang

**Affiliations:** 1 Department of Exercise and Health Science, National Taipei University of Nursing and Health Science, No. 365, Ming-te Rd., Peitou Dist., Taipei, 112303, Taiwan, 886 228227101 ext 7721

**Keywords:** exergame, functional fitness, sport technology, physical activity, stampede training

## Abstract

**Background:**

With the growing prevalence of aging populations, improving the health and quality of life of older adults has become a critical concern globally. In this context, sports technology presents promising applications. The exergame-based training mat—an electronic exercise technology—integrates gamification with diverse training designs, offering a safe and engaging approach to promoting health and well-being in older adults.

**Objective:**

This study aims to examine the impact of a novel exergame-based mat training program on community-dwelling older adults, particularly evaluating its effectiveness in enhancing physical activity levels, quality of life, and functional fitness. The primary end point was exploratory, focusing on the feasibility and effectiveness of the exergame-based training program on physical fitness, physical activity, and quality of life.

**Methods:**

This randomized parallel-designed study enrolled 32 older adults aged 60-80 years from Taipei City. Participants were randomly assigned to either the experimental or control group. The experimental group underwent a 10-week exergame-based mat training program, consisting of 2 sessions per week (70 minutes per session), using gamified group-based exercise training. The control group maintained their usual daily activities. Pre- and postintervention assessments were conducted using the International Physical Activity Questionnaire (IPAQ), the World Health Organization Quality of Life Brief Version (WHOQOL-BREF), the Senior Fitness Test, and the AFAscan fitness assessment.

**Results:**

The experimental group demonstrated significantly increased overall and high-intensity physical activity levels (*P*=.04; mean difference [MD] 439, 95% CI 28-914; *d*=0.72). Quality of life significantly enhanced across the physical (*P*=.01; *r*=0.53), psychological (*P*=.02; *r*=0.52), and social (*P*=.02; *r*=0.50) domains of the WHOQOL-BREF. Furthermore, functional fitness parameters, including upper limb muscular strength (*P*=.007; MD 5.33, 95% CI 1.59-9.07; *d*=1.06), lower limb muscular strength (*P*=.01; MD 4.73, 95% CI 1.15-8.32; *d*=0.98), core strength (*P*<.001; MD 13.1, 95% CI 7.90-18.2; *d*=1.89), lower limb flexibility (*P*=.008; MD 6.47, 95% CI 1.82-11.1; *d*=1.04), dynamic balance (*P*=.03; MD –0.72, 95% CI −1.36 to −0.07; *d*=0.84), static balance (*P*=.005; MD 15.1, 95% CI 5.01-25.3; *d*=1.12), agility (*P*=.001; MD 32.6, 95% CI 15.6-49.6; *d*=1.44), and cardiorespiratory endurance (*P*=.04; MD 9.73, 95% CI 0.37-19.1; *d*=0.78), showed significant enhancements with the exergame-based mat training program. There were no adverse events observed during the study.

**Conclusions:**

In this exploratory trial, the exergame-based mat training program produced medium-to-large improvements (Cohen *d* ranging from 0.72 to 1.89) across physical activity, quality of life, and functional fitness domains. Although the precision of the CIs varied, the consistent direction of effects supports a meaningful impact of the intervention. These findings suggest that exergame-based mat training programs may serve as a practical community health promotion strategy, potentially preventing age-related frailty and enhancing independence and well-being among older adults.

## Introduction

As global populations age, the increasing proportions of older adults pose various risks and challenges for individuals and families, and for social, economic, and health care systems. The need for preventive health care strategies to reduce, delay, or prevent chronic diseases while maintaining independent living has become important [1]. The age-related loss of muscle mass, strength, and function is influenced by aging, disease, and other lifestyle factors. Physical inactivity, malnutrition, unhealthy habits, and chronic diseases further increase the risk of sarcopenia, leading to decreased survival rates, prolonged hospitalization, falls, fractures, metabolic disorders, and cognitive impairment, finally diminishing the quality of life [2]. In addition, aging is associated with sensory decline and cognitive deterioration, severely influencing independence, self-care abilities, and overall well-being among older adults. However, increased physical activity and structured exercise interventions can mitigate the adverse effects of aging, thereby improving the quality of life [3].

Regular exercise interventions enhance physical fitness in older adults, reduce fall risks, improve mobility, and prevent certain conditions, including cardiovascular diseases, cognitive impairment, osteoporosis, and muscle weakness, all of which enhance the quality of life [4]. Moreover, age has a significant impact on the quality of life among older adults, whereas engagement in physical activity contributes to enhancing their quality of life, thereby promoting healthier lifestyles and greater personal independence [5]. Compared with other types of exercise-based interventions, previous studies have indicated that resistance-based interventions are the most effective for enhancing physical activity and strength; however, age and sex may act as confounding factors influencing these outcomes [6]. Therefore, in the context of global aging, maintaining health-related physical fitness is essential for delaying muscle loss, preventing falls and injuries, and mitigating chronic diseases, all of which improve the quality of life. Exercise programs, consequently, play an indispensable role in promoting healthy aging.

With rapid technological advancements, active video games and exergames provide older adults with diverse training options beyond traditional fitness programs. Wii Fit (Nintendo) exercise games enhance balance functions in community-dwelling older adults while improving their self-perceived confidence, with high acceptance rates among older populations [7]. Based on the experiences and needs of older adults, exergames are perceived as enjoyable and engaging. In addition to promoting physical activity, they can help alleviate feelings of loneliness and are not constrained by specific usage conditions. The development of well-designed exergames may therefore provide valuable support for this population [8]. Compared with traditional exercise methods, Kinect-based (Microsoft Corporation) exergames have demonstrated superior efficacy in improving executive function, cognitive performance, and functional fitness [9]. In addition, exergames enhance motivation for physical activity, enabling older adults to experience exercise benefits engagingly and enjoyably [10]. Compared with conventional exercise training, exergames not only improve physical fitness but also provide cognitive and psychological benefits. Therefore, exergames are a promising strategy for health promotion and wellness among the aging population.

Recent meta-analyses have consistently reported that exergame interventions in older adults produce small-to-moderate improvements in balance, mobility, and engagement [11,12]. A more recent systematic review further highlighted that home-based exergames are generally well accepted and improve mobility-related outcomes such as the Timed Up and Go test [13]. These findings underscore the potential of gamified training to enhance functional fitness; however, most previous protocols have focused on platforms such as Wii or Kinect, often emphasizing balance or general mobility with limited attention to agility. In contrast, the present exploratory trial used a novel “stampede” agility-focused exergame delivered twice weekly via the mat exercise, integrating multidirectional stepping, reaction-based tasks, and functional movements. The specified exergames, such as Wii and Kinect, can be integrated with motion-sensing technology and have demonstrated applications beyond entertainment, extending to therapy, rehabilitation, and health promotion. However, as both Wii and Kinect have been discontinued, their long-term applicability is limited, underscoring the need for developing updated platforms tailored for diverse populations. However, assessing movement quality remains a critical concern in implementing exergames, because inappropriate compensatory movement patterns may impact both effectiveness and safety [14].

Therefore, this study seeks to introduce an exercise mat training program led by a certified coach and to investigate its effectiveness within a 10-week comprehensive training regimen designed within the exergame framework. This protocol design addresses agility and dynamic balance more directly than the majority of exergame interventions previously synthesized in meta-analyses, thereby contributing new evidence on the scope and feasibility of agility-oriented exergaming for older adults. We aimed to evaluate functional fitness in older adults using both the standard Senior Fitness Test and the sport technology–based AFAscan fitness assessment. Furthermore, we intended to explore overall physical activity levels and quality of life. We hypothesized that a well-designed exergame program incorporating novel exercise mat training may enhance not only physical fitness but also physical activity and quality of life.

## Methods

### Experimental Design

In this exploratory trial, we recruited older adults aged 60-80 years from the University Social Responsibility program at National Taipei University of Nursing and Health Sciences University (Taipei City), and used a randomized parallel design. A total of 32 participants were randomly assigned to either the experimental (n=16) or control group (n=16) through simple randomization using a random number table, with an allocation ratio of 1:1. The personnel (research assistant) responsible for enrolling participants and those (principal investigator) managing the random allocation sequence were distinct, ensuring that the allocation process remained blinded and minimizing the potential for allocation bias. A pre- and postintervention design was implemented. The intervention consisted of a 70-minute exergame-based mat training session conducted twice weekly for 10 consecutive weeks. Each session included a 10-minute warm-up, approximately 45 minutes of primary training, and a 15-minute cooldown and stretching routine (Table 1). Participants completed the International Physical Activity Questionnaire (IPAQ) and the World Health Organization Quality of Life questionnaire (WHOQOL-BREF, Taiwanese Version) before and after the intervention. In addition, the Senior Functional Test and AFAscan fitness assessment (Scanleader, Scanleader Technologies Co. Ltd.) were conducted. Participants in the control group were instructed to maintain their usual daily activities without any additional structured exercise intervention during the study period. A no-intervention control condition was selected to provide a natural reference for functional fitness and quality-of-life changes without training. This approach allowed us to better isolate the effects of the exergame-based mat training and is consistent with previous exercise trials involving older adults.

**Table 1.** Training schedule, objectives, and equipment of the exergame-based mat program.

Week	Warm-up	Main course (training objectives)	Cooldown	Indicator light mode^a^	Equipment
1	Stretching exercise and built-in program^b^	Flexibility, agility, upper and lower limb strength, core muscles, cardio	Stretching	All light on, circle light on, 4 corners	Medicine ball
2	Stretching exercise and built-in program^b^	Agility, balance, lower limb strength	Stretching	All light on, circle light on, 4 corners	Medicine ball
3	Stretching exercise and built-in program^b^	Agility, cardio, upper and lower limb strength, flexibility	Stretching	All light on, circle light on, 4 corners	Medicine ball
4	Stretching exercise and built-in program^b^	Lower limb strength, cardio, flexibility	Stretching	All light on, circle light on, 4 corners, 2 lines, diamond	Elastic band, medicine ball
5	Stretching exercise and built-in program^b^	Upper and lower limb strength, balance, core muscles	Stretching	All light on, circle light on, 4 corners, 2 lines, diamond	Elastic band, medicine ball, kettlebell
6	Stretching exercise and built-in program^b^	Core muscles, upper and lower limb strength, agility	Stretching	All light on, circle light on, 4 corners, 2 lines, diamond	Elastic band, medicine ball, kettlebell
7	Stretching exercise and built-in program^b^	Core muscles, upper and lower limb strength, cardio	Stretching	All light on, circle light on, 4 corners, 2 lines, diamond	Elastic band, medicine ball, kettlebell
8	Stretching exercise and built-in program^b^	Upper and lower limb strength, core muscles, agility	Stretching	All light on, circle light on, 4 corners, 2 lines, diamond	Elastic band, medicine ball, kettlebell
9	Stretching exercise and built-in program^b^	Upper and lower limb strength, core muscles, balance	Stretching	All light on, circle light on, 4 corners, 2 lines, diamond	Elastic band, medicine ball, kettlebell, balance cushion
10	Stretching exercise and built-in program^b^	Upper and lower limb strength, lower limb explosive strength, balance, core muscles	Stretching	All light on, circle light on, 4 corners, 2 lines, diamond	Elastic band, medicine ball, kettlebell, balance cushion

a The lighting modes of the mat serve as visual cues, guiding participants to step on specific areas to turn off the lights as part of the exercise protocol.

b The built-in program comprises five regular exercise games (lights out game, jumping jacks, reactions, two-way jumping jacks, and whack-a-mole).

A randomized parallel-controlled clinical trial was conducted. A total of 32 eligible participants were randomly assigned to 2 groups: control group and experimental group. The participants completed questionnaires (the IPAQ and WHOQOL-BREF), along with physical fitness assessments (Senior Fitness Test and AFAscan Fitness Assessment), before and after the intervention. Finally, 30 participants completed all experimental procedures required for data analysis.

### Exergame-Based Mat Training Program

**Figure 1.**
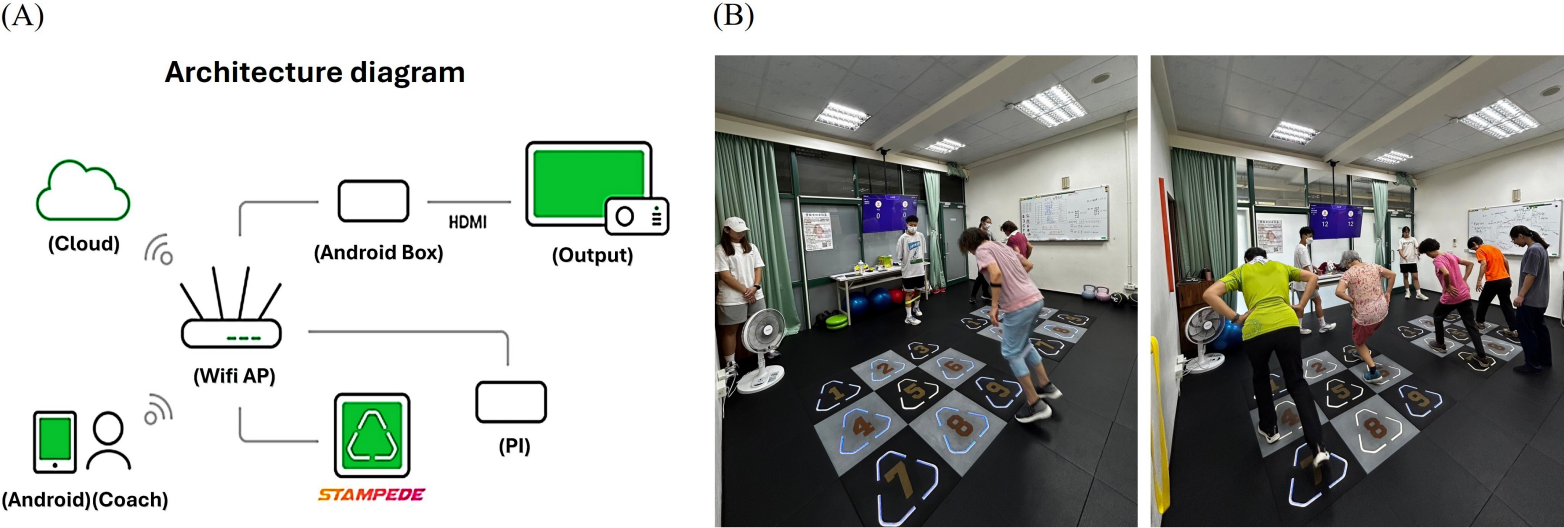
Stampede exercise mat system. (A) A central router facilitates data transmission among a cloud server, user interface devices (such as smartphones), and an analysis unit. The system highlights seamless data flow, emphasizing connectivity, data processing, and user interaction. (B) Stampede exercise mats use lighting and sound effects to guide participants through exergame implementation in the National Taipei University of Nursing and Health Sciences I203 laboratory. In addition, the system can be integrated with other exercise equipment to enhance fitness benefits while incorporating elements of enjoyment and gamification.

**Figure 2.**
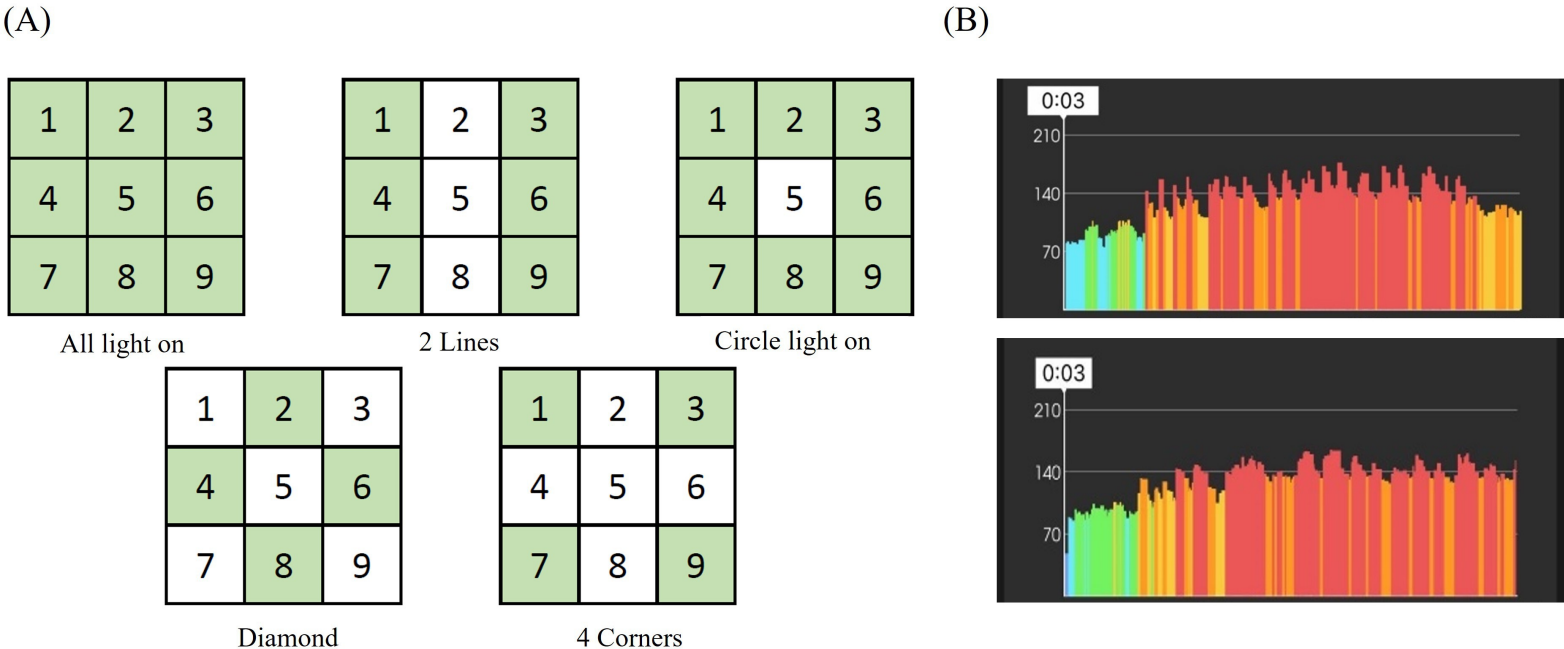
Exergame-based mat training modes and intensity monitoring. (A) This study used 5 lighting modes (all lights on, circle on, 4 corners, 2 lines, and diamond) for designated training protocols or in combination with other exercise equipment. (B) Heart rate was continuously monitored throughout the program.

The mat-based exercise system (Stampede, SHENNONA Co., Ltd) facilitates exercise training by using electronic light and sound effects to guide participants through various exercises, with intensity progressively increasing from moderate to high levels. This system facilitates customized movement programming tailored to specific training objectives, and its architectural diagram is presented in Figure 1A. The training program is divided into 2 phases: the first phase focuses on fundamental movements, whereas the second phase introduces more advanced exercises. With progress, additional exercise equipment is incorporated, along with increased sets, duration, movement variations, and training intensity. Training sessions can be conducted in a simple progressive manner or a game-competitive format (Figure 1B). Throughout the training, participants wore a heart rate monitor (ALATHEC Obeat1) that recorded their exercise intensity and heart rate fluctuations (Figure 2B). Exercise intensity was indicated by lighting colors, corresponding to specific percentages of the maximum heart rate (HRmax) as follows: blue (0%-50% HRmax), cyan (50%-60% HRmax), green (60%-70% HRmax), yellow (70%-80% HRmax), orange (80%-90% HRmax), and red (>90% HRmax). However, the exercise intensity and frequency were adjusted based on participants’ adaptation. This study encompassed 5 different lighting modes, namely all light on, circle on, 4 corners, 2 lines, and diamond (Figure 2A). Each mode was paired with different training protocols and movement patterns, supplemented by training equipment, such as medicine balls, resistance bands, kettlebells, and balance pads. This combination enhanced engagement while simultaneously promoting functional fitness, including muscular strength, muscular endurance, aerobic capacity, flexibility, agility, balance, and core strength. Detailed training protocols are provided in Multimedia Appendix 1. All participants achieved a 100% attendance rate, and adverse events were observed by qualified specialists. All training sessions were supervised and instructed by certified fitness instructors.

### Body Composition

InBody 270 (Biospace) was used as the body composition analyzer. It is a noninvasive bioelectrical impedance analysis device, which estimates body composition by transmitting a low-level electrical current through the body and measuring resistance. It provides accurate assessments of BMI, body fat percentage, obesity analysis, skeletal muscle mass, and visceral fat levels, serving as a comprehensive tool for evaluating body composition. The detailed procedures have been described previously [15].

### Senior Fitness Test

In older adults, various components of functional fitness were evaluated using standardized tests. Aerobic endurance was assessed through the 2-minute step test, whereas upper and lower limb muscular endurance were measured using the arm curl test and chair stand test, respectively. Lower limb flexibility was evaluated with the sit-and-reach test, and shoulder flexibility was assessed using the back-scratch test. In addition, static balance was measured using the one-leg standing test, and dynamic balance was evaluated using the 8-foot up-and-go test. Detailed procedures, devices, and manipulation have been described previously [16]. These senior functional tests are cost-effective and convenient for assessing functional fitness in older adults. They are suitable for independently living individuals aged 60-90 years and above, encompassing numerous fitness levels, ranging from frailty to high fitness [17]. All certified assessors, independent from the research team, conducted the Senior Fitness Assessments and AFAscan fitness assessment in a single-blind manner.

### AFAscan Fitness Assessment

The AFAscan leader facility was used to assess various components of physical fitness, including lower body explosiveness, core strength, flexibility, agility, balance, and endurance. It used gyroscopes (positioned on the chest and both thighs) and a force platform to precisely and objectively measure all physical performance parameters.

#### Lower Body Explosiveness

Lower body explosiveness was evaluated using a countermovement jump with an arm swing. Participants jumped to the highest extent while attempting to land in the same position on the force mat from which they took off. During the airborne phase, they maintained full extension of their hip, knee, and ankle joints to prevent any additional flight time caused by leg bending. Flight time was recorded as the primary measurement reference.

#### Core Strength

Core strength was measured using the bent-knee sit-up exercise. Participants lay on their backs with their knees flexed and feet positioned 12 inches from their buttocks. To ensure stability, a partner could assist by holding their feet in place. Participants conducted curl-ups, raising their upper bodies to at least 35°, and completed the fullest possible repetitions within 60 seconds.

#### Flexibility

Flexibility was assessed using the standing toe touch exercise. Participants stood with their feet together, toes pointing forward on the force mat. They bent forward at the hips, attempting to touch their fingertips to that of their toes without bending their knees. They held the position for 3 seconds to allow for angle measurement, which served as the flexibility assessment outcome.

#### Agility

Agility was assessed using the quick feet test. Participants alternately raised their feet in rapid succession, taking the shortest possible steps. They tried completing the maximum number of repetitions within 15 seconds.

#### Balance

Balance was assessed using the single-leg stand test. Participants raised their preferred foot and placed it against the inner thigh of the standing leg. They maintained a steady center of gravity within the center of a designated circle. A higher score was achieved by remaining stable within the center for a longer duration, with a maximum assessment time of 30 seconds.

#### Endurance

Endurance was assessed using the high-knee test. Participants drove their right knee toward their chest and quickly returned it to the ground, continuously alternating with the opposite knee. Arm swinging was permitted to aid movement. Participants raised their knees to an indicated angle >50° and completed the maximum number of repetitions within 30 seconds.

### Questionnaires

Physical activity levels were measured using the IPAQ, which evaluates walking, moderate-intensity, and vigorous-intensity activities over the past 7 days. The IPAQ has shown good test-retest reliability (Spearman ρ=0.76-0.94) and acceptable internal consistency (Cronbach *α*=0.80), with evidence of concurrent validity when compared against accelerometer data [18,19]. The Taiwanese version of the IPAQ was used, specifically the long-form Taiwan Physical Activity Survey Questionnaire, which consists of 26 items encompassing four domains: work, household activities, transportation, and leisure activities [20].

In addition, the Taiwanese version of the WHOQOL-BREF—a short version of the World Health Organization Quality of Life Assessment questionnaire—was used. This version consists of 28 items, encompassing 4 domains: physical health, psychological well-being, social relationships, and environmental factors. The WHOQOL-BREF has demonstrated acceptable internal consistency, with Cronbach α ranging from 0.66 to 0.84 across domains, and satisfactory construct validity when tested in different populations [21,22]. This shortened and adapted version serves as a valid alternative to the long-form WHOQOL questionnaire used in Taiwan [22].

### Eligibility Criteria

The inclusion criteria were as follows: (1) older adults (aged 60‐80 years) with no musculoskeletal injuries in the past month and capable of participating in training activities, (2) completion of the Physical Activity Readiness Questionnaire (PAR-Q) and eligibility for moderate-intensity exercise intervention, and (3) ability to adhere to the exercise frequency required by the study and independently commute to the training site. The exclusion criteria were as follows: (1) individuals with musculoskeletal disorders, joint diseases, unstable cardiovascular conditions, or cognitive impairments, as well as those with psychiatric disorders based on questionnaire evaluation, and (2) individuals whose PAR-Q responses indicate potential risks or concerns.

### Statistical Analyses

Descriptive statistics and chi-square tests were used to analyze the anthropometric and sociodemographic characteristics of all participants. The normality of all dependent variables was evaluated using Kolmogorov-Smirnov tests to ensure the appropriate selection of parametric and nonparametric analyses. To examine significant between- and within-group differences, nonparametric methods (Wilcoxon signed-rank test and Mann-Whitney *U* test) and parametric methods (mixed 2-way analysis of variance, unpaired 2-tailed *t* tests, and paired *t* tests) were applied. Two participants withdrew before completing posttest assessments and thus provided only baseline data. Because no postintervention outcome data were available for these individuals, no imputation was applied, and analyses were performed using complete cases. Consequently, a total of 15 participants in the experimental group and 15 participants in the control group were included in the final statistical analysis. All statistical analyses were performed using SPSS software (version 22; IBM Corp), with statistical significance set at *P*<.05. Effect sizes (*d*) were calculated as Cohen *d* and interpreted according to conventional benchmarks: small (0.2), medium (0.5), and large (0.8) [23]. The 95% CIs were reported alongside all effect size estimates to enhance interpretability.

### Ethical Considerations

This study was reviewed and approved by the Fu Jen Catholic University Institutional Review Board (IRB # C112161) per ethical guidelines for human research. Before participation, all participants were informed about the study procedures and precautions, and written informed consent was obtained. All acquired data will be analyzed and disseminated in deidentified format to ensure participant confidentiality. Study participants will receive individualized physical fitness assessment reports and monetary compensation consisting of US $32.77 (exchange rate: US $1=30.5 TWD) for transportation expenses. To ensure participant safety, all training sessions were supervised by qualified specialists, who continuously monitored for potential and unexpected adverse events. Any adverse event would have been documented immediately, managed with appropriate first aid, and referred for medical evaluation if necessary.

## Results

### Anthropometric Characteristics of Participants

**Figure 3.**
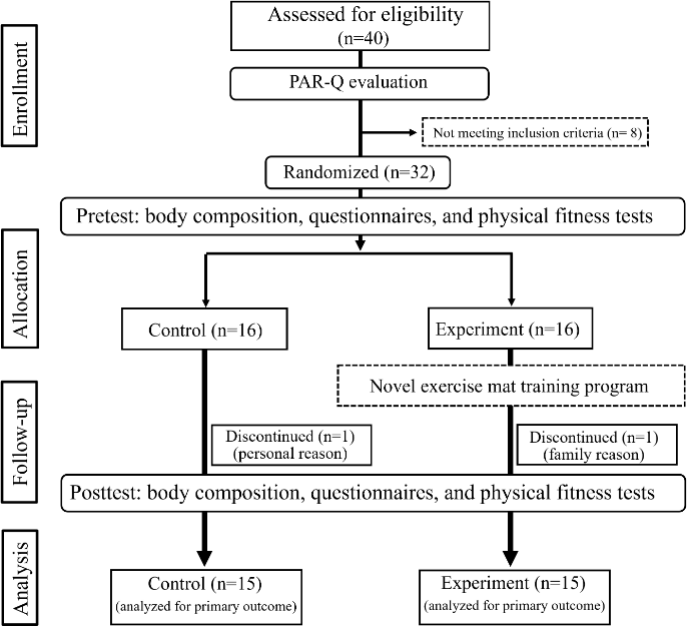
Study flow diagram. PAR-Q: Physical Activity Readiness Questionnaire.

The experimental procedure is illustrated in Figure 3. This study recruited 32 participants, with 16 allocated to each of the experimental and control groups. However, one participant from each group withdrew from the study because of personal reasons. Finally, 15 participants in both groups were analyzed for primary outcomes. The participants in the experimental group were paired and engaged in the training activities through alternating and cooperative interactions. Heart rate monitoring during the training indicated fluctuations characteristic of high-intensity interval training (Figure 2B). In addition, no significant differences were observed in sex, age, height, weight, systolic, diastolic pressure, and resting heart rate (*t*_28_=0.065‐1.121; all *P*>.05) before the intervention (Table 2).

**Table 2.** Basal anthropometric characteristics.

Characteristics	Control (n=15)	Experiment (n=15)	Mean difference (95% CI)	Effect size (Cohen *d)*
Sex (male/female), n	2/13	2/13	—^a^	—
Age (years), mean (SD)	67.7 (4.4)	67.8 (3.9)	0.13 (–2.96 to 3.22)	0.03
Height (cm), mean (SD)	157.7 (3.5)	157.8 (5.7)	0.11 (–3.43 to 3.65)	0.02
Weight (kg), mean (SD)	50.2 (9.4)	55.4 (13.8)	5.26 (–3.63 to 14.2)	0.44
Systolic pressure (mm Hg), mean (SD)	121.6 (17)	127.8 (15)	6.13 (–6.17 to 18.4)	0.37
Diastolic pressure (mm Hg), mean (SD)	75.4 (10)	77.0 (9.2)	1.60 (–5.47 to 8.67)	0.17
Resting heart rate (bpm), mean (SD)	81.6 (11)	85.4 (12)	3.87 (–4.83 to 12.6)	0.33

a Not applicable.

### Effects of the Exergame-Based Mat Program on Physical Activity

Pre- and postintervention results of the Physical Activity Scale indicated a significant main effect of time on total physical activity levels (Table 3). After the intervention, activity levels significantly increased in the experimental group, with significant within-group differences. Therefore, the mat exercise program effectively enhanced overall physical activity in older adults. For vigorous physical activity, the main effect of time (*F*_1,28_=13.9; *P*=.001) and interaction effect (*F*_1,28_=11.9; *P*=.002) were observed. The experimental group demonstrated a significant within-group increase in total metabolic equivalents (MET) (*t*_14_=2.88; *P*=.01; mean difference [MD] 1266, 95% CI 325-2206; *d*=0.74) and vigorous activity (*t*_14_=7.37; *P*<.001; MD 950, 95% CI 674-1227; *d*=1.90), indicating that the intervention effectively promoted physical activity. In addition, after the intervention, the experimental group demonstrated significantly higher vigorous activity levels than the control group (*t*_28_=1.97; *P*=.04; MD 439, 95% CI 28-914; *d*=0.72), supporting the effectiveness of the exergame-based mat program. However, no significant main effects or interaction effects were observed for moderate-intensity activity, walking, or sedentary behavior (*F*_1,28_=0.583‐1.889; *P*>.05). Thus, the intervention exerted a relatively lower impact on moderate- and low-intensity physical activities.

**Table 3.** Effects of the exergame-based mat training program on physical activity levels in older adults: effect sizes and 95% CIs.

Physical activity intensity	Control (MET^a^-min/week^b^), mean (SD)		Experiment (MET-min/week^b^), mean (SD)		Mean difference (95% CI)	Effect size, Cohen *d*
	Pre	Post	Pre	Post		
Total MET	2866 (1540)	3328 (2045)	2052 (1650)	3318 (1440)^c^	–9.67 (–1332 to 1313)	0.01
Vigorous intensity	789 (692)^d^	828 (762)	317 (417)	1267 (406)^c^^,^^d^	439 (28 to 914)	0.72
Moderate intensity	752 (945)	1252 (1029)	560 (782)	757 (620)	–495 (–1131 to 141)	0.58
Walking	1153 (862)	1076 (715)	1112 (817)	1164 (732)	85.8 (–456 to 627)	0.12
Sitting (work)	297 (139)	211 (119)	206 (158)	230 (182)	19.3 (–96 to 134)	0.13
Sitting (holiday)	277 (134)	239 (111)	278 (157)	282 (144)	43.3 (−52 to 140)	0.34

a MET: metabolic equivalents.

b MET·min·wk⁻¹

c Indicates significant within-group differences.

d Indicates significant between-group differences.

### Effects of Exergame-Based Mat Program on Quality of Life

The WHOQOL-BREF assessment evaluated overall health and health satisfaction, along with four domains: physical health, psychological health, social relationships, and environment (Table 4). Concerning physical health, the experimental group demonstrated significant differences both within (*P*=.02) and between (*P*=.01; Hodges-Lehmann median difference=1.2; 95% CI 0.6-2.3; *r*=0.53) groups. Specifically, significant within-group improvements were observed in pain and discomfort (*P*=.02), dependence on medical aids (*P*=.002), mobility (*P*=.008), and daily activities (*P*=.002). In addition, daily activities (*P*=.002) and work capacity (*P*=.006) showed a significant between-group difference in the experimental group, compared with the control group. Concerning psychological health, the experimental group exhibited a significantly higher overall score than the control group (*P*=.02; Hodges-Lehmann median difference=2.7, 95% CI 1.3-4; *r*=0.52). Significant between-group differences were observed in self-esteem (*P*=.02) and negative feelings (*P*=.001), with negative feelings showing significant differences within (*P*=.02).

**Table 4.** Effects of the exergame-based mat training program on quality of life in older adults with effect sizes and 95% CIs.

Dimensions	Control, mean (SD)		Experiment, mean (SD)		Hodges-Lehmann, median difference (95% CI)	Effect size (*r*)
	Pre	Post	Pre	Post		
Overall health	3.4 (0.6)	3.3 (0.5)	3.6 (0.5)	4 (0.5)^a^	1 (0 to 2)	0.64
Health satisfaction	3.3 (0.7)	3.0 (0.6)	3.4 (0.6)	3.7 (0.5)^a^	1 (0 to 2)	0.65
Domains						
Physical health^b^	13.3 (1.3)	14.3 (1.8)	13.5 (0.9)	16.1 (1.6)^a^^,^^c^	1.2 (0.6 to 2.3)	0.53
Pain and discomfort	3.3 (0.5)	3.5 (0.9)	3.0 (0.5)	4.1 (0.8)^c^	1 (0 to 2)	0.35
Dependence on medical substance and medical aids	3.3 (0.9)	3.3 (0.8)	3.3 (0.5)	4.1 (0.6)^c^	0 (–1 to 1)	0.17
Energy and fatigue	3.5 (0.5)	3.3 (0.6)	3.4 (0.5)	3.6 (0.5)	0 (1 to 1)	0.29
Mobility	3.5 (0.6)	3.9 (0.5)	3.5 (0.5)	4.2 (0.7)^c^	0 (–1 to 1)	0.28
Sleep and rest	3.2 (1.1)	3.1 (0.9)	3.5 (0.7)	3.3 (0.8)	0 (–1 to 1)	0.07
Activities and daily living	3.3 (0.6)	3.5 (0.6)	3.3 (0.5)	4.3 (0.5)^a^^,^^c^	1 (0 to 2)	0.56
Work capacity	3.9 (0.5)	3.5 (0.5)	4.1 (0.7)	4.1 (0.5)^a^	1 (0 to 2)	0.50
Psychological health^b^	13.8 (1.9)	13.4 (2.0)	14.3 (1.7)	15.3 (1.6)^a^	2.7 (1.3 to 4)	0.52
Positive feeling	3.5(0.6)	3.3 (0.6)	3.5 (0.6)	3.7 (0.7)	1 (0 to 2)	0.34
Spirituality, religion, and personal beliefs	3.7 (0.7)	3.7 (0.6)	3.7 (0.9)	3.8 (0.7)	0 (–1 to 1)	0.14
Thinking, learning, memory, and concentration	3.5 (0.7)	3.1 (0.7)	3.8 (0.7)	3.5 (0.5)	0 (–1 to 1)	0.30
Bodily image and appearance	3.7 (0.6)	3.5 (0.6)	3.9 (0.7)	4 (0.8)	1 (0 to 2)	0.31
Self-esteem	3.9 (0.6)	3.5 (0.6)	3.7 (0.6)	4.1 (0.5)^a^	1 (0 to 2)	0.42
Negative feelings	3.3 (0.6)	3.1 (0.7)	3.4 (0.9)	3.9 (0.5)^a,^^c^	1 (0 to 2)	0.64
Social relationship^b^	13.4 (2.1)	12.8 (1.9)	14.4 (2.6)	14.5 (1.5)^a^	2 (1 to 3)	0.50
Personal relationship	3.9 (0.6)	3.2 (0.6)^c^	3.9 (0.9)	3.7 (0.6)	1 (0 to 2)	0.38
Sexual activity	3.2 (1.1)	3.1 (0.8)	3.4 (0.7)	3.2 (1.1)	0 (–1 to 1)	0.23
Social support	3.7 (0.6)	3.3 (0.6)^c^	3.6 (0.7)	3.9 (0.5)^a^	1 (0 to 2)	0.56
Being respected	3.7 (0.6)	3.3 (0.5)^c^	3.8 (1.1)	3.7 (0.7)	1 (0 to 2)	0.31
Environment^b^	15.1 (1.7)	14.6 (1.8)	15.7 (1.7)	15.8 (1.4)	1.8 (0.9 to 2.7)	0.39
Freedom, physical safety, and security	3.7 (0.8)	3.6 (0.6)	4.1 (0.6)	4 (0.5)	0 (–1 to 1)	0.30
Physical environment	3.6 (0.8)	3.4 (0.5)	3.6 (1.0)	3.4 (0.6)	0 (–1 to 1)	0.06
Financial resource	3.8 (0.7)	3.6 (0.7)	4.2 (0.7)	3.8 (0.9)	1 (0 to 2)	0.19
Opportunities for acquiring new information and skills	3.9 (0.6)	3.9 (0.6)	4.0 (0.5)	4.2 (0.6)	1 (0 to 2)	0.26
Participation and opportunities for recreation/leisure activities	3.8 (0.7)	3.7 (0.5)	4.2 (0.8)	3.8 (0.7)	1 (0 to 2)	0.15
Home environment	3.7 (1.1)	3.8 (0.6)	3.9 (0.7)	3.9 (0.5)	1 (0 to 2)	0.44
Health and social care: accessibility and quality	4.3 (0.7)	4.3 (0.5)	4.3 (0.5)	4.2 (0.5)	1 (0 to 2)	0.40
Transport	4.3 (0.6)	4.3 (0.5)	4.3 (0.5)	4.3 (0.6)	1 (0 to 2)	0.40
Eating	4.1 (0.6)	3.8 (0.7)	3.9 (0.6)	4.0 (0.8)	1 (0 to 2)	0.15

a Indicates significant between-group differences.

b Raw domain scores for the World Health Organization Quality of Life Brief Version are transformed to a 4 to 20 score according to guidelines (Total score of the participant in a specific domain×4) divided by (Total number of items in the domain).

c Indicates significant within-group differences.

Concerning social relationships, the experimental group demonstrated significantly higher overall scores than the control group (*P*=.02; Hodges-Lehmann median difference 2, 95% CI 1-3; *r*=0.50). Furthermore, the experimental group exhibited significantly higher scores in social support than the control group (*P*=.003). In contrast, regarding the environment, no significant within- or between-group differences were observed in overall scores or individual items, indicating that the intervention did not substantially affect this aspect of quality of life.

### Effects of Exergame-Based Mat Program on Senior Fitness

Table 5 summarizes the results for systolic blood pressure, diastolic blood pressure, and heart rate. No significant interaction effects were observed between group and time (*F*_1,28_=0.583‐1.889; all *P*>.05). In addition, no significant main effect of group was observed (*F*_1,28_=1.505‐2.181; all *P*>.05), except for a significant main effect of time on diastolic blood pressure (*F*_1,28_=4.350; *P*=.04). In the control group, systolic blood pressure and heart rate did not change, whereas diastolic blood pressure significantly reduced after the intervention. In the experimental group, systolic blood pressure, diastolic blood pressure, and heart rate showed no significant between- or within-group differences.

**Table 5.** Effects of the exergame-based mat training program on Senior Fitness Test outcomes in older adults with effect sizes and 95% CIs.

Variables	Control, mean (SD)			Experiment, mean (SD)			Mean difference (95% CI)	Effect size (Cohen *d*)
	Pre	Post	Gain score	Pre	Post	Gain score		
Systolic pressure (mm Hg)	121.6 (17)	116.6 (17)	–5.1 (15)	127.8 (15)	126.2 (16)	–1.6 (8.1)	9.60 (–2.83 to 22)	0.58
Diastolic pressure (mm Hg)	75.4 (10)	70.6 (8.4)^a^	–4.9 (6.7)	77 (9.2)	76.1 (7.6)	–1 (8.5)	5.47 (–0.54 to 11.5)	0.68
Resting heart rate (bpm)	81.6 (11)	78.1 (6.3)	–3.5 (11)	85.4 (12)	85.1 (13.5)	–0.4 (7.6)	7 (–0.89 to 14.9)	0.66
Arm curl test (times)	17.7 (5)	17.4 (5.3)	–0.3 (2.9)	17.7 (2.9)	22.7 (4.6)^a^^,^^b^	5.1 (3.9)^b^	5.33 (1.59 to 9.07)	1.06
Chair stand test (times)	23.7 (5.7)	22 (4.7)	–1.6 (4.1)	23.6 (4.5)	26.8 (4.8)^a^^,^^b^	3.2 (3.8)^b^	4.73 (1.15 to 8.32)	0.98
Chair sit-and-reach (cm)	7.8 (4.4)	5.8 (6.8)	–2 (4.1)	7.9 (6)	12.3 (5.5)^a^^,^^b^	4.4 (3.6)^b^	6.47 (1.82 to 11.1)	1.04
Back-scratch test (cm)	6.6 (4.2)	6.6 (5.1)	–0.1 (2.6)	5.8 (3.1)	6.4 (4.2)	0.5 (3.5)	–0.18 (–3.67 to 3.31)	0.04
One-leg standing test (s)	27.8 (5.5)	26.7 (7.2)	–1.1 (3.7)	26 (7.3)	28.7 (5.2)	2.7 (5.2)	2 (–2.67 to 6.67)	0.32
8-foot up-and-go test (s)	4.3 (0.6)	4.4 (0.6)	0.2 (0.3)	4.3 (0.5)	3.7 (1.1)^b^	–0.6 (1.1)^b^	–0.72 (–1.36 to –0.07)	0.84
2-minute step test (times)	109 (11)	109 (12)	– 0.3 (7.7)	107 (8.8)	119 (13)^a^^,^^b^	12 (10)^b^	9.73 (0.37 to 19.1)	0.78

a Indicates significant within-group differences.

b Indicates significant between-group differences.

For muscular endurance of both upper and lower limbs, a significant interaction effect was observed between time and group (*F*_1,28_=18.1, *P*<.001; *F*_1,28_=10.91, *P*=.003, respectively). Specifically, upper limb muscular endurance in the experimental group was significantly higher in the posttest compared with the pretest (*t*_14_=4.92; *P*<.001; MD 5.07, 95% CI 2.80-7.27; *d*=1.27), and significantly higher compared with the control group (*t*_28_=2.92; *P*=.007; MD 5.33, 95% CI 1.59-9.07; *d*=1.06). Moreover, the gain score for upper limb endurance was significantly higher in the experimental group than in the control group (*t*_28_=4.26; *P*<.001; MD 5.4, 95% CI 2.80-7.99; *d*=1.55). Similarly, lower limb muscular endurance was significantly higher in the experimental group after the intervention compared with before the intervention (*t*_14_=3.24; *P*=.006; MD 3.2, 95% CI 1.08-5.31; *d*=1.55) and the control group (postintervention values; *t*_28_=2.70; *P*=.01; MD 4.73, 95% CI 1.15-8.32; *d*=0.98). The gain score for lower limb endurance was significantly higher in the experimental group than in the control group (*t*_28_=3.31; *P*=.003; MD 4.80, 95% CI 1.83-7.77; *d*=1.21).

No significant interaction effect was observed between time and group for upper limb flexibility (*F*_1,28_=0.284; *P*=.59). No significant between- or within-group differences were observed for upper limb flexibility after the intervention. However, a significant interaction effect was observed for lower limb flexibility (*F*_1,28_=20.71; *P*<.001). However, lower limb flexibility in the experimental group was significantly higher after the intervention than before the intervention (*t*_14_=4.74; *P*<.001; MD 4.41, 95% CI 2.41-6.40; *d*=1.04). Postintervention values were significantly higher than those in the control group (*t*_28_=2.85, *P*=.008, MD 6.47, 95% CI 1.82-11.1; *d*=1.04). In addition, the gain score for lower limb flexibility was significantly higher in the experimental group than in the control group (*t*_28_=4.55; *P*<.001; MD 6.45, 95% CI 3.54-9.36; *d*=1.66).

Regarding balance performance, no significant interaction effect was observed for static balance between time and group (*F*_1,28_=1.308; *P*=.29), with no significant between- or within-group differences. Nonetheless, a significant interaction effect was observed for dynamic balance (*F*_1,28_=6.646; *P*=.02). Despite no significant within-group difference in the experimental group before and after the intervention, the postintervention time was significantly lower than that in the control group (*t*_28_=−2.29; *P*=.03; MD -0.72, 95% CI −1.36 to −.07; *d*=0.84). Furthermore, the gain score for dynamic balance was significantly lower in the experimental group than in the control group (*t*_28_=−2.58; *P*=.02; MD −0.77, 95% CI −1.38 to −0.16; *d*=0.94).

For aerobic endurance, a significant interaction effect was observed between time and group (*F*_1,28_=14.027; *P*=.001). In the experimental group, postintervention aerobic endurance was significantly higher than preintervention endurance (*t*_14_=4.58; *P*<.001; MD 12, 95% CI 6.38-17.6; *d*=1.18). Moreover, postintervention values were significantly higher than those in the control group (*t*_28_=2.13; *P*=.04; MD 9.73, 95% CI 0.37-19.1; *d*=0.78). In addition, the gain score for aerobic endurance was significantly higher in the experimental group than in the control group (*t*_28_=3.75; *P*=.001; MD 12.3, 95% CI 5.59-19.1; *d*=1.66).

### Effects of Exergame-Based Mat Program on AFAscan Fitness Assessment

No significant interaction effect was observed for explosiveness, along with no significant between- or within-group differences (*F*_1,28_=0.169; *P*=.68) (Table 6). For core strength, a significant interaction effect was observed between time and group (*F*_1,28_=14.027; *P*=.001). In the experimental group, postintervention core strength was significantly higher than preintervention core strength (*t*_14_=3.77; *P*=.002; MD 8.14, 95% CI 3.47-12.8; *d*=1). Moreover, postintervention values were significantly higher than those in the control group (*t*_28_=5.18; *P*<.001; MD 13.1, 95% CI 7.90-18.2; *d*=1.89). In addition, the gain score for core strength was significantly higher in the experimental group than in the control group (*t*_28_=2.49; *P*=.02; MD 6.69, 95% CI 1.15-12.2; *d*=1.04).

**Table 6.** Physical fitness outcomes evaluated by the AFAscan assessment before and after the exergame-based mat training program with effect sizes and 95% CIs.

Physical fitness variables	Control, mean (SD)			Experiment, mean (SD)			Mean difference (95% CI)	Effect size (Cohen *d*)
	Pre	Post	Gain score	Pre	Post	Gain score		
Vertical jump (cm)	15.8 (3)	16.7 (4.1)	0.9 (2.9)	16.9 (4.5)	18.2 (3.5)	1.3 (2.5)	1.53 (–1.32 to 4.39)	0.40
Sit-up (times)	11.9 (7)	13 (5.5)	1.5 (4.1)	18.1 (8.8)	26.3 (8.1)^a^^,b^	8.1 (8)^a^	13.1 (7.90-18.2)	1.89
Toe-touch test (degree)	125 (16)^a^	112 (18)^b^	–13.4 (13)	113 (12)	125 (17)^a^^,b^	12.7 (11.2)^a^	13.3 (0.34-26.3)	0.77
Agility (times)	57.5 (28)	61.1 (24)	3.7 (25)	59.5 (29)	93.7 (21)^a^^,b^	34.1 (28)^a^	32.6 (15.6-49.6)	1.44
Balance test (scores)	37.3 (12)	35.5 (15)	–1.8 (15)	39.6 (12)	50.6 (12)^a^^,b^	11 (15)^a^	15.1 (5.01-25.3)	1.12
Endurance test (times)	69 (17)	65 (12)	–3.6 (14)	76 (20)	92 (15)^a^^,b^	17 (15)^a^	27 (16.6-37.4)	1.95

a Indicates significant between-group differences.

b Indicates significant within-group differences.

Significant interaction effects were observed for flexibility, agility, balance, and endurance (*F*_1,28_=5.27‐34.078; all *P*<.05). Postintervention fitness characteristics in flexibility (*t*_28_=2.10; *P*=.05; MD 13.3, 95% CI 0.34-26.3; *d*=0.77), agility (*t*_28_=3.93; *P*=.001; MD 32.6, 95% CI 15.6-49.6; *d*=1.44), balance (*t*_28_=3.06; *P*=.005; MD 15.1, 95% CI 5.01-25.3; *d*=1.12), and endurance (*t*_28_=5.34; *P*<.001; MD 27, 95% CI 16.6-37.4; *d*=1.95) were significantly improved in the experimental group, compared with the control group. Moreover, significant within-group differences were observed in the experimental group, with significantly higher gain scores than in the control group.

### Effects of Exergame-Based Mat Program on Body Composition

Regarding body composition (Table 7), no significant interaction effects were observed for body weight, BMI, skeletal muscle mass, body fat mass, or body fat percentage (*F*_1,28_=0.179‐0.347; all *P*>.05) between the control and experimental groups. In addition, no significant differences were observed in gain scores between groups. After the intervention, the experimental group exhibited an increasing trend in lower limb muscle mass, whereas the control group exhibited a minor decline in lower limb muscle strength. However, these changes were not statistically significant in terms of gain scores. Therefore, the intervention exerted a limited impact on the overall body composition, segmental muscles, and fat distribution.

**Table 7.** Effects of exergame-based mat training on body composition in older adults: effect sizes and 95% CIs.

Body composition variables	Control, mean (SD)			Experiment, mean (SD)			Mean difference (95% CI)	Effect size (Cohen *d)*
	Pre	Post	Gain score	Pre	Post	Gain score		
Body weight (kg)	50.2 (9.4)	50.1 (9.6)	–0.013 (1.3)	55.4 (13.8)	55.4 (13.7)	–0.013 (1.1)	5.62 (–3.59 to 14.1)	0.44
Body mass index	20.1 (3.6)	20.1 (3.7)	0 (0.5)	22.1 (4.1)	22.2 (4)	0.1 (0.4)	2.02 (–0.89 to 4.94)	0.52
Skeleton muscle (kg)	20.1 (2.8)	20.1 (3)	0.04 (0.5)	21.6 (4.7)	21.6 (4.7)	0.05 (0.7)	1.49 (–1.48 to 4.47)	0.37
Body fat mass (kg)	12.7 (6.2)	12.6 (6.2)	–0.12 (1.2)	15.4 (7.5)	15.5 (7.2)	0.14 (1.2)	2.93 (–2.11 to 7.97)	0.44
Body fat percent (%)	24.1 (8.6)	23.8 (8.6)	–0.31 (1.9)	26.7 (7.6)	26.9 (6.8)	0.11 (1.9)	3.10 (–2.73 to 8.93)	0.40
Segmental lean analysis (kg)								
Right arm	1.6 (0.4)	1.5 (0.4)	–0.098 (0.2)	1.9 (0.6)	1.8 (0.7)	–0.063 (0.1)	0.35 (–0.07 to 0.77)	0.62
Left arm	1.6 (0.4)	1.5 (0.4)	–0.117 (0.3)	1.8 (0.6)	1.8 (0.6)	–0.407 (0.1)	0.34 (–0.06 to 0.74)	0.62
Trunk	15.4 (2.7)	14.6 (3.8)	–0.740 (1.9)	17.1 (3.8)	16.9 (3.8)	–0.173 (0.7)	2.25 (–0.59 to 5.08)	0.59
Right leg	5.7 (0.8)	5.4 (1.3)	–0.277 (0.9)	6 (1.3)	6.1 (1.1)	0.072 (0.2)	0.67 (–0.28 to 1.65)	0.53
Left leg	5.7 (0.7)	5.4 (1.2)	–0.312 (0.9)	5.7 (1.8)	6 (1.3)	0.333 (1)	0.66 (–0.27 to 1.58)	0.53
Segmental fat analysis (kg)								
Right arm	0.9 (0.4)	0.8 (0.4)	–0.03 (0.1)	1 (0.6)	1.1 (0.6)	0.033 (0.1)	0.23 (–0.14 to 0.61)	0.47
Left arm	0.9 (0.4)	0.9 (0.4)	–0.04 (0.1)	1.1 (0.6)	1.1 (0.6)	0.013 (0.1)	0.23 (–0.14 to 0.61)	0.47
Trunk	5.7 (3.5)	5.5 (3.3)	–0.27 (0.8)	7.3 (4.2)	7.3 (4)	–0.033 (0.7)	1.83 (–0.92 to 4.59)	0.50
Right leg	2.1 (0.9)	2.1 (0.9)	–0.03 (0.3)	2.4 (1)	2.5 (1)	0.08 (0.2)	0.44 (–0.25 to 1.13)	0.48
Left leg	2.1 (0.9)	2.1 (0.9)	–0.03 (0.2)	2.4 (1)	2.5 (1)	0.09 (0.2)	0.43 (–0.26 to 1.11)	0.47

### Pearson Correlation Between Functional Fitness in the Senior Fitness Test

The magnitude of correlations was interpreted according to Cohen conventions: small (0.10-0.29), moderate (0.30-0.49), and large (≥0.50). Positive values indicate direct associations, while negative values indicate inverse associations. Pearson correlation analysis of physical fitness assessments in older adults (Table 8) suggested a significantly moderate positive correlation between upper and lower limb muscular endurance (*r*=0.502; *P*<.05). In addition, a significantly low positive correlation between upper limb muscular endurance and lower limb flexibility was observed (*r*=0.377; *P*<.01). However, no significant correlations were observed between upper limb muscular endurance and either static, dynamic balance, or aerobic endurance. Lower limb muscular endurance demonstrated a significantly low to moderate positive correlation with both cardiorespiratory endurance (*r*=0.488; *P*<.01) and lower limb flexibility (*r*=0.386; *P*<.01), whereas a significantly moderate negative correlation with dynamic balance (*r*=−0.6; *P*<.01) was observed. In addition, lower limb flexibility exhibited a significantly moderate positive correlation with static balance (*r*=0.402; *P*<.01). Therefore, the intervention effectively enhanced multiple aspects of physical fitness, underscoring the interplay among fitness components.

**Table 8.** The Pearson correlation coefficients and 95% CIs for functional fitness as evaluated by the Senior Fitness Test.

Senior physical fitness		Arm curl	Chair stand	Sit-and-reach	One-leg standing	8 ft up-and-go test	2-minute step
Arm curl							
*r* (95% CI)		1	0.502 (0.17 to 0.73)	0.377 (0.02 to 0.65)	–0.063 (–0.39 to 0.33)	–0.063 (–0.39 to 0.33)	0.189 (–0.28 to 0.44)
*P* value		—^a^	.005	.04	.74	.74	.32
Chair stand							
*r* (95% CI)		0.502 (0.17 to 0.73)	1	0.386 (0.05 to 0.66)	0.072 (–0.33 to 0.39)	–0.600 (–0.79 to –0.31)	0.488 (0.16 to 0.72)
*P* value		*.* 005	—	.04	.71	<.001	.006
Sit-and-reach							
*r* (95% CI)		0.377 (0.02 to 0.65)	0.386 (0.05 to 0.66)	1	0.402 (0.05 to 0.66)	–0.149 (–0.43 to 0.29)	0.242 (–0.23 to 0.47)
*P* value		.04	.04	—	.03	.43	.20
One-leg standing							
*r* (95% CI)		–0.063 (–0.39 to 0.33)	0.072 (–0.33 to 0.39)	0.402 (0.05 to 0.66)	1	–0.195 (–0.44 to .27)	0.009 (–0.35 to 0.36)
*P* value		.74	.71	.03	—	.30	.96
8 ft up-and-go test							
*r* (95% CI)		–0.063 (–0.39 to 0.33)	–0.600 (–0.79 to –0.31)	–0.149 (–0.43 to 0.29)	–0.195 (–0.44 to 0.27)	1	–0.207 (–0.44 to 0.27)
*P* value		.74	<.001	.43	.30	—	.27
2-minute step							
*r* (95% CI)		0.189 (–0.28 to 0.44)	0.488 (0.16 to 0.72)	0.242 (–0.23 to 0.47)	0.009 (–0.35 to 0.36)	–0.207 (–0.44 to 0.27)	1
*P* value		.32	.006	.20	.96	.27	—

a Not applicable.

## Discussion

### Principal Findings

Integrating exergames, motion-sensing technologies, and sports-based innovations is essential for the future development of physical fitness interventions. Well-structured training programs not only promote exercise participation and overall health across diverse populations but also enhance the regularity and acceptability of fitness behaviors. Accordingly, the present 10-week mat exercise program was designed based on an exergame strategy, incorporating a variety of exercise equipment to achieve multiple training objectives under coach supervision. The sessions used a 2-person exergame-based mat system that featured turn-taking, cooperative, and competitive game-based activities. In terms of functional fitness, significant improvements were observed in muscular endurance, lower limb flexibility, static and dynamic balance, core strength, agility, and aerobic endurance. Moreover, physical activity levels and quality of life—particularly aspects related to physical health, psychological well-being, and social relationships—were also enhanced as a result of this novel exergame-based mat training intervention.

Traditional community-based exercise programs, including aerobic, resistance, balance, and stretching training, have been shown to improve daily physical activity and aerobic capacity in older adults [24]. However, for those with mild disabilities, such interventions primarily enhance specific fitness components such as lower limb strength and flexibility without significantly affecting overall activity levels [25]. Alternative approaches, such as stationary cycling-based high-intensity interval training (HIIT), effectively increase moderate-to-vigorous activity and energy expenditure [26]. Similarly, motion-based exergames like the Nintendo Wii and Xbox Kinect increase energy expenditure and improve upper limb endurance, physical activity, and psychological well-being, particularly among sedentary or mobility-limited older adults [27,28]. These findings suggest that program design, intervention duration, and participant health status critically shape outcomes. Building on this evidence, our exergame-based mat training program integrated exergaming elements, progressive intensity, HIIT mode, and group-based sessions to provide a comprehensive physical activity experience, yielding significant gains in vigorous activity and functional fitness.

Regarding quality of life, a study [29] on Kinect-based exergames reported significant improvements in physical and psychological health, aligning with our findings. Both the exercise mat program and Kinect training enhanced mobility, reduced pain, and improved daily functioning. Psychologically, the exercise mat training boosted self-esteem and reduced negative emotions. Multicomponent exercise training has also been shown to enhance executive functioning in older adults, contributing to improved quality of life [30]. In addition, exergaming programs significantly improve self-efficacy in older adults, particularly by fostering confidence in maintaining long-term exercise participation [31]. This underscores the role of gamification and social interaction in overcoming psychological barriers and building confidence in physical activity. A systematic review further confirmed the effectiveness of exergames in promoting mental health and socialization among older adults, though identifying the most effective types of exergames remains an area for future research [32]. The exergame-based mat training in this study was designed to achieve multiple fitness goals by incorporating enjoyable, electronic elements to evoke positive emotions. Group-based sessions fostered social interaction and reduced loneliness, while the progressive intensity design introduced appropriate challenges to enhance self-efficacy. Together, these factors contributed to improved physical function and psychological adaptability in older adults.

Our findings also align with meta-analyses demonstrating that active video games improve balance, lower limb strength, and cardiorespiratory fitness in older adults, though effects on body composition and flexibility remain limited [33,34]. In the present study, the exergame-based mat training yielded similar results, with significant improvements in balance, flexibility, muscular strength, and aerobic capacity, despite no significant changes in body composition. Older adults without a history of falls exhibited superior performance, highlighting the role of static balance, lower limb strength, and flexibility in reducing fall risk in this population [35]. Previous studies consistently highlight the critical role of lower limb strength in older adults. Greater lower limb strength has been linked to enhanced static and dynamic balance [36,37], greater functional independence and self-care ability [38], as well as higher quality of life [39]. Together, these findings emphasize that maintaining lower limb strength is fundamental not only for mobility and fall prevention but also for preserving autonomy and overall well-being in later life. Our findings suggest that older adults who participated in the exergame-based mat training demonstrated functional improvements across functional fitness indicators, which may have implications for fall prevention, independence, and overall quality of life.

Exergame interventions provide multifaceted benefits for older adults, including improvements in strength, balance, flexibility, and cardiorespiratory endurance [40]. Although our mat-based exergame training did not reduce body fat, it effectively enhanced upper limb and core strength, aerobic endurance, agility, and balance, thereby reducing fall risk, consistent with prior mat-based studies [41]. Similar to Bacha et al [42], who reported superior cardiorespiratory fitness gains with Kinect-based exergames compared to conventional therapy, our findings further underscore the potential of exergame-based approaches for improving aerobic capacity and overall function. Given evidence linking physical fitness to fall frequency [43], the observed improvements in balance and muscular strength highlight clinically meaningful outcomes. Moreover, parallels with HIIT research indicate that the present exergame training program may facilitate favorable physiological adaptations, such as improved metabolic and cardiovascular health, while mitigating age-related decline [44-46].

This study has several limitations. Long-term quantitative tracking using accelerometry was not implemented, and participants’ dietary caloric intake was neither controlled nor documented, limiting adjustments for physical activity and body composition effects. Upper limb flexibility requires further exploration through targeted exercises and equipment design. In addition, the benefits observed cannot be conclusively attributed to the program, and the lack of an attention-matched intervention for the control group may have introduced bias in self-reported outcomes, such as physical activity and quality of life. However, the inclusion of objective measures (eg, mat-based kinetics and standardized timed tests) provides supportive evidence for the observed effects. As an exploratory trial, the exergame-based training program demonstrated diverse benefits in physical fitness, physical activity, and quality of life. Future research should refine training intensity and modes, include appropriate comparator groups, and further investigate the program’s physiological and therapeutic impacts to enhance its applicability for diverse older adult populations.

### Conclusion

The implementation of exergame-based mat training positively influenced functional fitness in older adults. These gains are likely to translate into improved performance of daily physical activities, including enhanced walking capacity, prolonged standing tolerance, greater movement flexibility, and increased independence in activities of daily living. Beyond physical benefits, participation in exergame-based mat training positively influenced psychological well-being by boosting self-confidence and self-esteem while promoting greater social participation. In addition, the integration of group-based exergame and interactive experiences enhanced the willingness and acceptance of older adults to engage in training. These findings highlight the feasibility and benefits of the program in improving functional fitness and overall quality of life among older adults.

## Supplementary material

10.2196/73474 Multimedia Appendix 1 Training protocol.

10.2196/73474 Checklist 1 CONSORT checklist.

## Abbreviations

HIIT: high-intensity interval training
HRmax: maximum heart rate
IPAQ: International Physical Activity Questionnaire
MD: mean difference
MET: metabolic equivalents
NTUNHS: National Taipei University of Nursing and Health Sciences
PAR-Q: Physical Activity Readiness Questionnaire
USR: University Social Responsibility
WHOQOL-BREF: World Health Organization Quality of Life Brief Version
