# Supplementary material for: A Novel Gamified Exercise Program Incorporating Stampede Training for Enhancing Functional Fitness, Physical Activity Levels, and Quality of Life in Community-Dwelling Older Adults: Randomized Parallel Exploratory Trial
Source: JMIR Serious Games. 2025 Nov 3;13:e73474. doi: 10.2196/73474 (PMC12582559; doi:10.2196/73474)
Supplement: Multimedia Appendix 1 [file games-v13-e73474-s001.pdf]

Physical Activity Main Course Taring Protocol							
**Week**	**Warm up**	**All Light on[#]**	**Circle Light On[#]**	**Two Lines[#]**	**4 corners[#]**	**Diamond[#]**	**Cool-down**
		1. Single person's feet jump and step to turn-off light	1. Two people stand on 5, turn off the light with one right foot/Two people stand 5, turn off the light with one left foot (change sides)		1. Two people sit after 2 and 8 positions respectively, straighten their feet and turn off the lights with hands.		
		2. Two people step to turn-off light with their feet (in turn)	2. One person is on 5 position and use the medicine ball to hit the light source to turn-off the light (in turn). Another person to pick up the ball and pass the ball to the person standing on the 5.		2. Two people sit after 2 and 8 positions, respectively, for Jumping Jacks movements to turn off light.		
**1**	5-10 Mins (with 5 Basic protocol[*])	3. Single person steps to turn-off light with one right foot (in turn)			3. Two people use their feet to alternatively jump left and right to turn off the lights.		10 Mins
		4. Single person steps to turn-off light with one left foot (in turn)			4. Single person performs plank pose in positions 1 (hands) and 7 (feet) and turn off the lights on positions 3 and 9 (in turn)		
		5. All Four Position to turn off the lights (in turn)					
		6. Two people turn off the lights together with both hands (Plank position).					

	5-10 Mins	1. Step on and turn off the lights in sequence with both feet (extinguishing order: 9-6-3-2-5-8-7-4-1).	1. Face forward and step two steps per square to turn off the lights (extinguishing order: 9-6-3-2-1-4-7-8).		1. In a seated position with legs extended straight, use both hands to turn off the lights.		
		2. Step on and turn off the lights in sequence with one foot.	2. Squat movement and step to turn off the lights.		2. Two people take turns using one foot as the pivot while the other foot quickly moves forward and backward to turn off the lights (switch pivot foot).		
**2**	5 Basic protocol	3. Use the right foot to step and jump to turn off the lights individually.	3. One person turns off the lights with one foot; the right foot steps on 3, 6, 9, and 8, while the left foot steps on 2, 1, 4, and 7.		3. Take two steps per square, moving forward and backward.		10 Mins
		4. Use the left foot to step and jump to turn off the lights individually.	4. Two people pass a medicine ball to each other and use it to turn off the lights, passing the ball after turning off each light.		4. Use one foot as the pivot while the other foot jumps forward and backward to turn off the lights (switch pivot foot).		
		5. Step on and turn off the lights randomly with both feet as fast as possible.	5. Two people in a quadruped position use their hands to turn off the lights.				
**3**	5-10 Mins	1. Use the tiptoe of both feet to step on the designated lights (order: 9-6-3-2-5-8-7-4-1).	1. Jump with both feet to turn off the lights.		1. In a seated position with legs extended straight, use both hands to turn off the lights.		10 Mins
	5 Basic protocol	2. Turn off the lights by performing jumping jacks.	2. Turning off light while walking in a squat position.		2. Use the maximum jumping range with both feet to turn off the lights.		

		3. One person uses a medicine ball to turn off the lights while the other passes the ball (in turns).<br>4. Perform the plank position supported by hands and use both hands to turn off the lights.	3. Use high-knee running to turn off the lights (two steps per square).		3. Take two steps per square for forward and backward movement.<br>4. Use one foot as the pivot while the other foot jumps forward and backward to turn off the lights (switch pivot foot).		
**4**	5-10 Mins<br>5 Basic protocol	1. Use the right foot to hop and turn off the lights (order: 9-6-3-2-5-8-7-4-1).<br>2. Use the left foot to hop and turn off the lights (order: 9-6-3-2-5-8-7-4-1).<br>3. Wear a mini band around the lower legs and step one square at a time to turn off the lights.<br>4. Wear a mini band around the forearms, assume a plank position, and use both hands to turn off the lights.	1. Jump with both feet to turn off the lights.<br>2. Wear a mini band around the lower legs and walk in a squat position to turn off the lights.<br>3. Jump with both feet to turn off the lights, returning to the centre after turning off each light.	1. Move forward with high-knee running and backward with two steps per square.<br>2. Two people perform jumping jacks to turn off the lights.<br>3. Hop side-to-side with one foot to turn off the lights (order: 9-7-6-4-3-1).	1. In a seated position with knees bent, two people hold a medicine ball and perform Russian twists to turn off the lights.<br>2. Two people use a medicine ball to turn off the lights alternatively.	1. Two people wear mini bands around their feet, fix the pivot foot, and use the other foot to turn off the lights.<br>2. Perform jumping jacks in lateral and front-back directions.<br>3. Jump with both feet to turn off the lights, returning to the centre after turning off each light (order: 2-4-6-8).	10 Mins

		5. Turn off the lights randomly at the fastest speed while moving in a backward position.					
5	5-10 Mins<br>5 Basic protocol	1. Place the large resistance band around the body, one person pulls the rope, and the other person jumps on one foot to turn off the light.	1. Hold the medicine ball in your hands, and turn off the light on one foot (right foot for positions 2369, left foot for positions 1478).	1. steps to forward to turn off the light with high knees for the 963 & 741, and then small steps to back 369 & 147. In this exercise for 2 persons.	1. Two people perform Russian twists while holding the medicine ball to turn off the light.	1. Place the mini band around the calves and use the right foot to turn off the light at positions 68, and the left foot to turn off the light at positions 24.	10 Mins
		2. Place the mini band around the calves, and quickly step one step at a time to turn off the light.	2.Hold the medicine ball in your hands, and turn off the light on one foot (right foot for positions 3698, left foot for positions 2147).	2. Two people use the medicine ball to turn off the light; after completing one row of lights, pass the ball to the other person to turn off another the lights.	2. Two people place the mini band around their forearms, perform a plank with hand support, and use both hands to turn off the light.	2. Two people hold the kettlebell and perform deep squats to turn off the light.	
		3. stand in the middle behind the 8 number, then go to the 8 positions to turn off the light for right to 9, left to 7, go for 5 then turn off the light for 6 and left for 4. Then go to middle position in the number 2 then turn off the light to the 3 or right, and 1 for left		3. Perform single-leg lateral jumps to turn off the light (order 9-7-6-4-3-1).	3. Hold the medicine ball and perform the highest possible jump with both feet.		
		4. Use both hands to turn off the light at positions 123, and use both feet to turn off the					

		light at positions 456789.					
**6**	5-10 Mins<br><br>5 Basic protocol	1. A large resistance band is placed around one person, who steps forward to turn off the light, while another person pulls the rope to increase resistance.<br>2. A large resistance band is placed around one person, who steps forward to turn off the light (speed-up), while another person pulls the rope to increase resistance.<br>3. Place the mini band around the calves and jump with both feet to turn off the light.<br>4. Stepping to turn off the light (order: 9-6-3-2-5-8-7-4-1)	1. Place the mini band around the calves, and walk with squat position to turn off the light.<br>2. Perform a plank with hand support and use both hands to turn off the light.	1.Hold the kettlebell with both hands and perform a deep squat to extinguish the light (standing sideways).<br>2. Use the medicine ball to alternately turn off the light from left to right, while the other person passes the ball.	1. Russian twist for ready position with hold the medicine ball and then hit the light using the ball to turn off the light number 9&7, and 1&3. When hit the one number the subject will do a crunch before hit or turn off another light/number.<br>2. Two people place the mini band around their forearms, perform a plank with hand support to turn off the light.<br>3. Hold the medicine ball overhead and perform a jump to turn off the light.	1. Place the mini band around the calves and fix one leg to turn off the light with another leg.<br>2. Two people use one leg to hook the kettlebell and turn off the lights.	10 Mins

7	5-10 Mins<br><br>3 Basic Protocol	1. Place the large resistance band around the body, one person pulls the rope, and the other person steps side-to-side in a lateral movement to turn off the light (switch sides).<br><br>2. Place the large resistance band around the body, one person pulls the rope, and the other person quickly steps to turn off the light (with increased resistance).	1. Sit on the floor, lean your body back, and use your hands to turn off the light.<br><br>2. Place the mini band around the forearms and use the right hand to turn off the light at positions 2369, and the left hand to turn off the light at positions 1478.<br><br>3. Place the mini band around the feet, raise one knee and move it forward to turn off lights (switch legs).	1.Hold the kettlebell with both hands and raise it to chest height, then perform a deep squat walk to extinguish the light.<br><br>2. Use the medicine ball to alternately turn off the light from left to right, while the other person passes the ball.	1. Russian twist for ready position with hold the medicine ball and then hit the light using the ball to turn off the light number 9&7, and 1&3. When hit the one number the subject will do a crunch before hit or turn off the another light/number.<br><br>2. Hold the medicine ball overhead and jump with both feet to turn off the light.	1. Jump with both feet to turn off light and return to 5 for next jump.<br><br>2. Step with both feet to turn off light and return to 5 for next jump.<br><br>3. Two people use one leg to hook the kettlebell and turn off the lights.	10 Mins

8	5-10 Mins	1. A large resistance band is placed around one person, who quickly jumps forward to turn off the light, while another person pulls the rope to increase resistance.	1. Hold the kettlebell and perform a deep squat walk to turn off the light.	1.Two people hook a kettlebell with one leg and perform the turning-off light.	1. One person holds a medicine ball and performs Russian twists, while the other person does sit-ups to turn off the light (pass the medicine ball between each other).	1. Place the mini band around the calves, fix the left foot, and jump with the right foot to turn off the light (switch legs).	10 Mins
		2. A large resistance band is placed around one person, who quickly steps forward to turn off the light with foot fire, while another person pulls the rope to increase resistance.	2. Place the mini band around the feet and perform a single-leg knee raise forward to turn off the light (switch legs).	2.Turning off the lights with Single-hand kettlebell	2. Hold the medicine ball overhead and jump with both feet to turn off the light.	2. Place the mini band around the calves, fix the axis foot, and jump to turn off the light.	
	3 Basic Protocol	3. Turning off all the lights with medicine ball (in turn)		3. Place the mini band around the forearms, perform a plank with hand support, and alternate hands to turn off the light.			
9	5-10 Mins	1. A large resistance band is placed around one person, who quickly steps forward to turn off the light, while another person pulls the rope to increase resistance.	1. Single-leg standing on a balance pad, the other leg single-leg turning off the lights (right leg 2369, left leg 1478)	1. Place a large resistance band around the body. One person pulls the band while the other performs side-to-side jumps.	1. Mini band around the calves and perform double-leg jumps (highest).	1. Wear a mini band around the lower legs, fix the left foot, and use the right foot to turn off the lights while facing backward.	10 Mins

	3 Basic Protocol	2. A large resistance band is placed around one person, who hops on one foot to turn off the light, while another person pulls the rope to increase resistance.<br><br>3. A mini band is placed around the forearms, hands in a plank position, and both hands are used to extinguish the light.	2. Double-leg jump<br><br>3. Mini band on forearms, and turning off lights (right hand on 2369, left hand on 1478)<br><br>4. Single-leg to turn off the lights with kettlebell	2. Hold a kettlebell in each hand and perform walking while in a squat position.	2. One person does Russian twists with a medicine ball, while the other does a sit-up for turning off light (medicine ball passed between them).<br><br>3. Sit on a balance pad and turn off lights with the hands.	2. Wear a mini band around the lower legs, fix the pivot foot, and use the other foot to jump and turn off the lights.<br><br>3. Tow way jumping Jacks	
**10**	5-10 Mins	1. Place a large resistance band around the body. One person pulls the band to increase resistance, while the other turns off the lights as quickly as possible.	1. Hold a kettlebell and perform double-leg jumps.	1. Place a large resistance band around the body. One person pulls the band while the other performs side-to-side jumps.	1. In a two-person elbow plank position, use hands to turn off the lights.	1. In a two-person side plank position, use hands to turn off the lights.	10 Mins

3 Basic Protocol	2. Two people use a mini resistance band on both hands arm. The hand movements to turn off the lights are 123, and the parallel jumping movements to turn off the lights are 456789.	2. Stand on a balance pad with one leg, while the other foot performs stepping movements to turn off the lights (right foot: 3698, left foot: 2147).	2. Place a large resistance band around the body. One person pulls the band while the other performs double leg jumps forward.	2. Place a mini resistance band around the lower legs, hold a medicine ball, and perform the highest possible double-leg jumps.	2. Use one foot to hook a kettlebell and turn off the lights.
	3. Hold a medicine ball and perform single-leg hops, alternating legs.	3. Place a mini resistance band around the lower legs and perform fast double-leg jumps facing forward.	3. Hold a kettlebell in each hand and perform walking while in a squat position.	3. One person performs Russian twists with a medicine ball, while the other does sit-ups and turns off the lights.	
		4. In a seated position, use a medicine ball to turn off the lights.	4. Use a medicine ball to powerfully hit the lights off from side to side.	4. Sit on a balance pad and use a medicine ball to turn off the lights.	
			5. Hold a medicine ball and perform side-to-side jumps.		

*, there are 5 regular exercise games (Lights Out Game, Jumping Jacks, Reactions, Tow way jumping Jacks, and Whack-a-Mole) in built-in program. #, The lighting modes of the mat serve as visual cues, guiding participants to step on specific areas to turn off the lights as part of the exercise protocol.
